# Relationship between mitochondrial DNA A10398G polymorphism and Parkinson's disease: a meta-analysis

**DOI:** 10.18632/oncotarget.20920

**Published:** 2017-09-15

**Authors:** Feifei Hua, Xiaona Zhang, Binghui Hou, Li Xue, Anmu Xie

**Affiliations:** ^1^ Department of Neurology, The Affiliated Hospital of Qingdao University, Qingdao, China; ^2^ Department of Rehabilitation, The Affiliated Hospital of Qingdao University, Qingdao, China

**Keywords:** Parkinson’s disease, mitochondrial DNA, gene polymorphism, meta-analysis

## Abstract

Many studies have researched the mitochondrial DNA (mtDNA) A10398G in Parkinson's disease (PD) to determine the association between mtDNA A10398G and PD, but the results of their research were not consistent. Therefore, we performed a meta-analysis to demonstrate the connection between mtDNA A10398G and the susceptibility of PD. We searched PubMed, Web of Science, Springer Link, EMBASE and EBSCO databases up to identify relevant studies. Through strict inclusion and exclusion criteria, at last, 9 studies (total 3381 cases and 2810 controls) were included in our meta-analysis. We used the STATA 12.0 statistics software to calculate the pooled odds ratios (ORs) and 95% confidence intervals (CIs) to evaluate the genetic association between mtDNA A10398G and the risk of PD. We performed subgroup analysis to clarify the possible roles of the mtDNA A10398G polymorphism in the aetiology of PD in different ethnicities. Our meta-analysis indicates that although there was no significant association between mtDNA A10398G and PD in the Asian population (G vs. A: OR = 1.090, 95% CI = 0.939–1.284, *P* = 0.242), in the Caucasian population the G allele of mtDNA A10398G mutations may be a potential protective factor of PD (G vs. A: OR = 0.699, 95% CI = 0.546–0.895, *P* = 0.005). Further well-designed studies with larger samples are needed to validate these results.

## INTRODUCTION

Parkinson's disease (PD), characterized by resting tremor, rigidity, slowness of movements and postural instability, is the second most common neurodegenerative disorder after Alzheimer's disease [1]. Although the cause of PD is still not completely known, it is already known that both genetic susceptibility factors and environmental factors could lead to the occurrence and the development of PD. According to one recent GWAS study related with PD [2], we know that a number of gene variants such as SNCA, LRRK2, PARK2 and GBA, have been shown to modulate the risk of PD. Additionally, mutations in a number of mitochondrial related genes, such as PARK6, PARK2, 12SrRNA, PINK1, POLG and DJ, have also been confirmed to be associated with PD [3, 4]. Among these genes, 12SrRNA was located in mitochondrial and the others were located in the nucleus. These genes participate in the coding of mitochondrial proteins required to build mitochondria and maintain the function of mitochondria [5].

Human mitochondria have their own DNA, containing 37 genes in a single chromosome [6]. Some genes located in the mitochondria encode subunits of enzymes that are components of the respiratory chain or ATP synthesis, thus playing a critical role in energy production. Since the brain constantly requires high amounts of energy consumption for maintenance of its functions, an alteration in the mtDNA functions may cause changes in the brain functions and brain disorders. The genetic polymorphism A10398G, due to an amino acid substitution from Thr to Ala, is located on the ND3 gene that encodes one subunits constituting Complex I [7]. In the substantia nigra and platelets of PD patients, the biochemical activity in the electron transport chain of complex I is impaired. Therefore, the 10398G allele could have an effect on the development of PD. In recent years, the polymorphism of mtDNA A10398G has been suggested as a protective factor in some studies [8–10] but not in others [11–15], and one study even indicated that it was a risk factor [16]. To help resolve the controversy over whether the mtDNA A10398G variant is associated with PD susceptibility, we conducted a meta-analysis according to the eligible case-control data from 9 reported studies.

## RESULTS

### Characteristics of eligible studies

**Figure 1:**
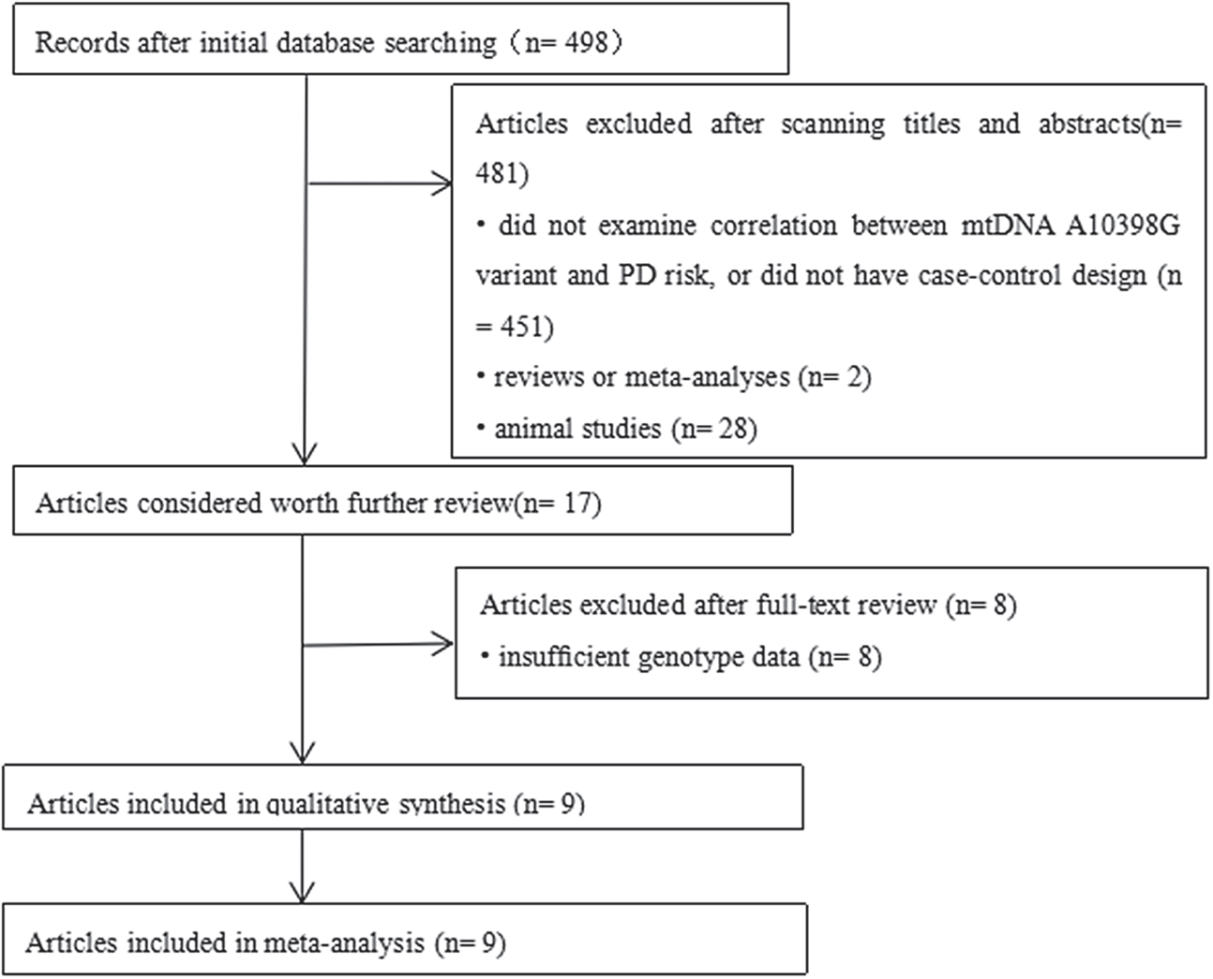
Flow diagram for publication selection in the present meta-analysis

The selection flow diagram and exclusion reasons for articles are described in Figure 1. First, 498 articles were searched as potential relevant articles through database searching. After scanning titles and abstracts, articles that did not examine the correlation between the mtDNA A10398G variant and PD risk or did not have a case-control design (*n* = 451) were excluded. In addition, reviews or meta-analyses (*n* = 2) and animal studies (*n* = 28) were excluded, and 17 potentially relevant articles were considered worth further reviewing. Then, through screening the full text, studies with no sufficient allele data (*n* = 8) were excluded, and 9 potentially relevant articles were identified. Finally, after estimating the quality of the eligible studies, 9 articles (including 9 clinical case-control studies with a total of 3381 PD cases and 2810 healthy individuals) met our inclusion criteria and were included in our meta-analysis. Among them, 6 studies’ subjects were Caucasian, and 3 studies’ subjects were Asian. In these studies, they used PCR or TaqMan as their genotyping method and the genotype frequencies of the controls were in HWE. The detailed characteristics of the included studies are described in Table 1, and the NOS scores of these articles are listed in Table 2.

**Table 1:** Characteristics of studies included in this analysis

ID	First author	year	Ethnicity	Genotyping method	Sample size (case/control)	Allele distribution (case/control)G A	HWE, *P* value	association
1	Cecilia Huerta	2006	Caucasian	PCR-sequencing	450/200	58/44 392/156	0.382	Had
2	Joanne Clark	2011	Caucasian	PCR-RFLP	376/173	84/44 292/129	0.571	Hadn’t
3	C. M. Chen	2007	Asian	PCR-RFLP	416/372	257/223 159/149	0.132	Hadn’t
4	Qiaohong Chu	2015	Asian	PCR-RFLP	322/332	186/168 136/164	0.573	Had
5	Cecilia Huerta	2005	Caucasian	PCR-RFLP	271/230	34/49 237/181	0.191	Had
6	D. Otaegui	2004	Caucasian	PCR-sequencing	40/64	9/10 31/54	0.068	Hadn’t
7	Joelle M. van der Walt	2003	Caucasian	Taqman	557/312	97/81 460/231	0.386	Had
8	Helen Latsoudis	2008	Caucasian	PCR-RFLP	224/383	43/78 181/305	0.368	Hadn’t
9	C. W. Liou	2016	Asian	SPCR	725/744	397/406 328/338	0.526	Hadn’t

PCR, polymerase chain reaction. PCR-RFLP, Polymerase Chain Reaction-Restriction Fragment Length Polymorphism.

SPCR, symmetric PCR. HWE, Hardy–Weinberg equilibrium. The *P* value of HWE determined by the χ^2^ test or Fisher's exact test in control groups.

**Table 2:** Quality assessment for the eligible studies according to NOS

	First author	Selection (stars)	Comparability (stars)	Exposure (stars)	Total Quality score	Refs.
1	Cecilia Huerta	4	2	1	7	8
2	Joanne Clark	3	2	1	6	12
3	C. M. Chen	4	2	1	7	13
4	Qiaohong Chu	4	2	1	7	16
5	Cecilia Huerta	4	2	1	7	9
6	D. Otaegui	4	2	1	7	11
7	Joelle M. van der Walt	3	2	1	6	10
8	Helen Latsoudis	4	2	1	7	14
9	C. W. Liou	3	2	1	6	15

NOS: Newcastle-Ottawa-Scale [32].

### Quantitative synthesis

**Figure 2:**
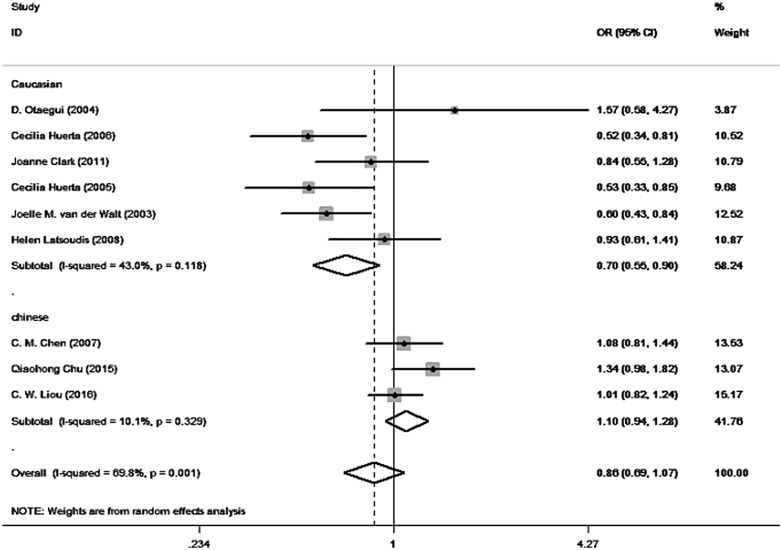
Forest plot for the relationship of mtDNA A10398G polymorphism and PD (G allele vs. A allele)

The results of our meta-analysis are summarized in Table 3. According to the I-squared value, heterogeneity was present in the allele model (I^2^ = 69.8%, *P* = 0.001), so the random effect model was selected. We also performed subgroup analysis stratified by ethnicity (in the Caucasian population: I^2^ = 43.0%, *P* = 0.118; in the Asian population: I^2^ = 10.0%, *P* = 0.329). According to our results, although no significant association between mtDNA A10398G polymorphism and PD was found when polling all populations overall(G vs. A: OR = 0.858, 95% CI = 0.686–1.074, *P* = 0.181) or in the Asian population (G vs. A: OR = 1.090, 95% CI = 0.939–1.284, *P* = 0.242), in the Caucasian population (G vs. A: OR = 0.699, 95% CI = 0.546–0.895, *P* = 0.005) the G allele of mtDNA A10398G mutations may be a potential protective factor of PD. The forest plot of mtDNA A10398G polymorphism in PD is shown in Figure 2.

**Table 3:** Meta-analysis of the association between the A10398G gene mutation and PD

Ethnicity	Fix-effect model			Random-effect model			Heteogeneity	Begg's test	
	OR	95% CI	*P*	OR	95% CI	*P*	I^2^	*Z*	*P*
Overall population	0.910	0.812–1.019	0.102	0.858	0.686–1.074	0.181	69.8%	0.52	0.602
Caucasian population	0.689	0.576–0.824	< 0.001	0.699	0.546–0.895	0.005	43.0%	< 0.01	1.000
Chinese population	1.094	0.945–1.267	0.231	1.098	0.939–1.284	0.242	10.1%	1.04	0.296

### Publication bias

**Figure 3:**
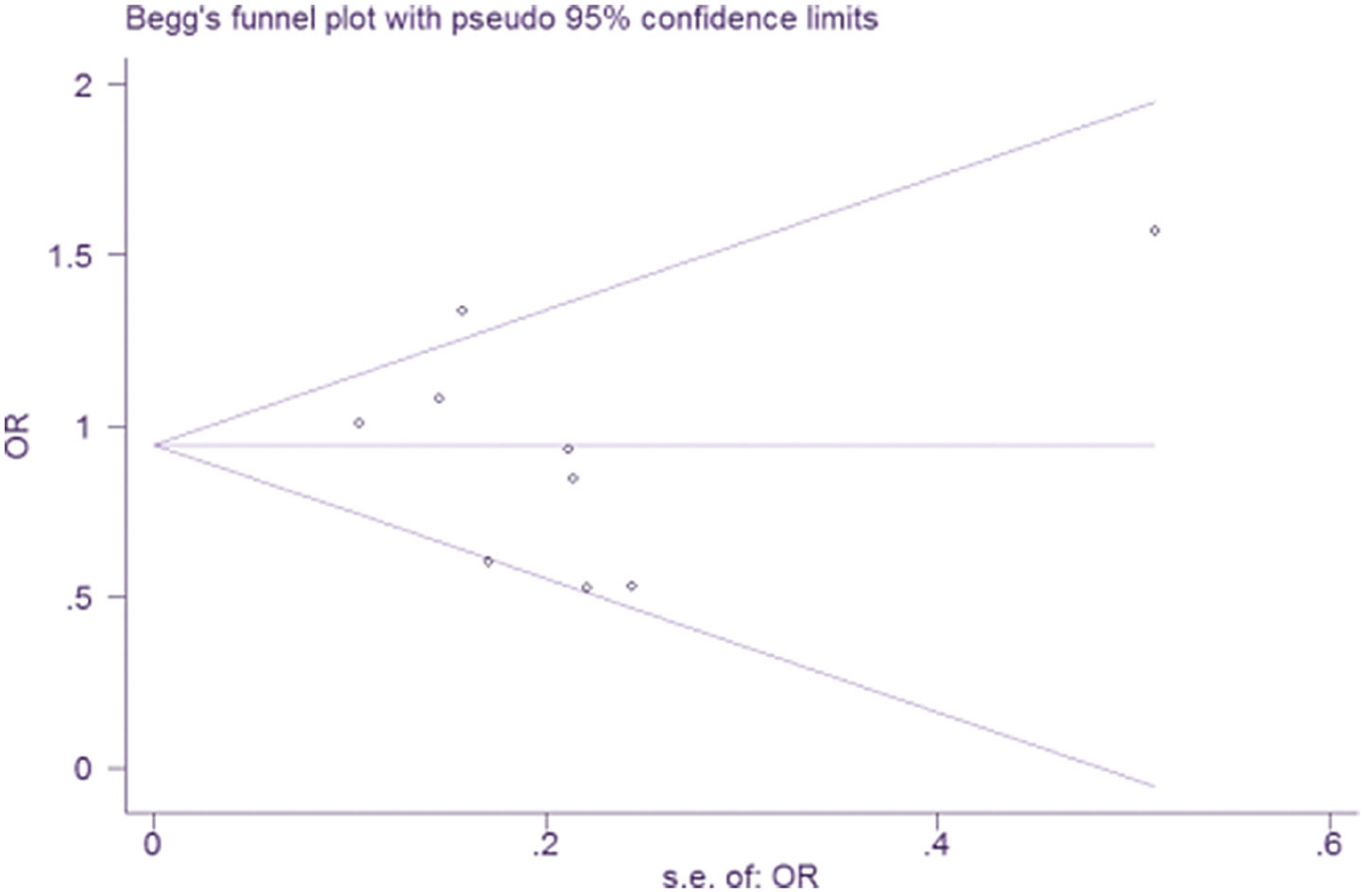
Begg's funnel plot for publication bias analysis for mtDNA A10398G polymorphisms

Begg's funnel plots and tests were used to estimate the publication bias. No publication bias was identified for the association between A10389G polymorphism and PD susceptibility (*P* = 0.602, Figure 3).

### Sensitivity analysis

**Figure 4:**
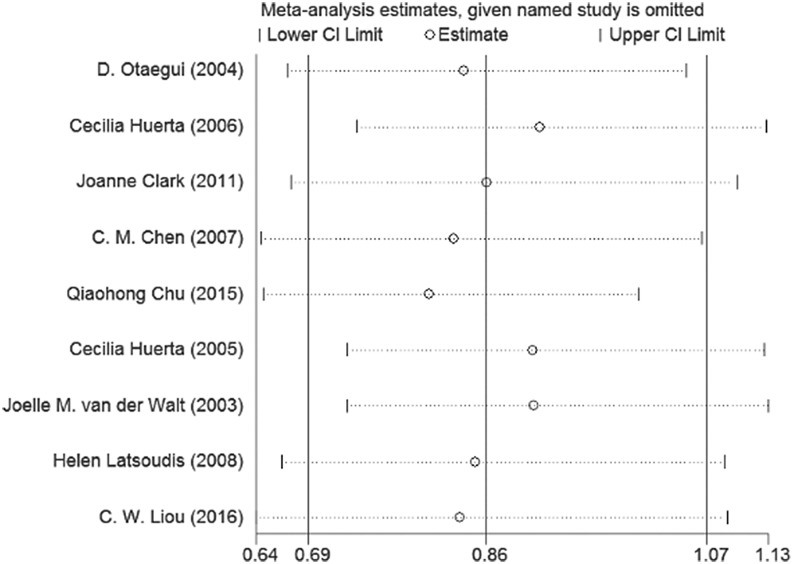
Sensitivity analysis of the the summary odds ratio coefficients on the association between mtDNA A10398G polymorphism and PD

We removed each study one by one every time the results showed no significant changes in the pooled OR and 95% CI in the allele model. This demonstrates that our meta-analysis is of high stability (Figure 4).

## DISCUSSION

As the second most common neurodegenerative disorder, PD is characterized by the loss of dopaminergic neurons in the substantia nigra (SN). We all know that although the aetiological factor of PD is still obscure, the genetic factor has been well confirmed to have a great contribution to the development of PD. Many studies have suggested that the dysfunction of mitochondria could be involved in the development of PD [17–19]. Additionally, mtDNA mutations have been reported to contribute to some neurodegenerative disorders, including Lewy body disease (LBD, mainly PD), Alzheimer's disease, amyotrophic lateral sclerosis (ALS) and multiple sclerosis (MS) [20–22]. However, the relationship between mtDNA A10398G and PD is still unclear. In the past several years, many studies have researched the relationship between the mtDNA A10398Gpolymorphism and the susceptibility of PD in different areas around the world, but their results are conflicting. Considering the contradictory conclusions that they reached and that most of these studies’ sample sizes were relatively small, a meta-analysis should be applied to further verify the association between mitochondrial DNA A10398G and PD.

In this meta-analysis, we included a total of 9 eligible case-control studies above. Among the 9 included studies, six were in the Caucasian population and three were in the Asian population. In a study based on a regional Caucasian population from Spain, with a total of 450 cases and 200 controls, the 10398G allele was found to decrease the risk of PD with an OR (95% CI) of 0.52 (0.34–0.81) [8]. In the study by Cecilia Huerta [9] with a total of 271 cases and 230 controls, the 10398G allele was also shown to decrease the risk of PD in the Spanish population with an OR (95% CI) of 0.53 (0.33–0.86) [9]. In the study by Joelle M. van der Walt [10], 10398G is strongly associated with a protective effect of PD in European with an OR (95% CI) of 0.53 (0.39–0.73). However, these results were not always consistent. Qiaohong Chu [16] detected an inverse association between A10398G polymorphism and the risk of PD. In a northern Chinese population,carrying the 10398G allele had a significantly increased risk of PD (OR = 1.30; 95% CI = 0.95–1.76; *P* = 0.013). After stratification by gender, the increased risk appeared to be more significant in females (OR = 1.91; 95% CI = 1.16–3.16; *P* = 0.001). Of note, such a statistically significant association between A10398G polymorphism and the risk of PD was not always duplicated in other studies. D. Otaegui [11] reported that no statistically significant correlation was identified in the general Caucasian population of Spain. However, Joanne Clark [12] observed that G10398G was not significantly associated with PD risk among the Caucasian population. Similarly, no association was detected in C. M. Chen's [13] case-control study comprising 416 cases and 372 controls. The frequencies of the G allele among cases and controls from both Helen Latsoudis's [14] case-control study (including 224 cases and 383 controls) and C. W. Liou's [15] study (including 725 cases and 744 controls) were similar to each other. For our meta-analysis, although the results support that individuals carrying the G allele did not exhibit an increased risk of PD as per the eligible studies, when compared with individuals carrying the A allele in the Asian population (G vs. A: OR = 1.090, 95% CI = 0.939–1.284, *P* = 0.242), the G allele of mtDNA A10398G polymorphism seems to be a potential protective factor of PD in the Caucasian population (G vs. A: OR = 0.699, 95% CI = 0.546–0.895, *P* = 0.005).

Mutation of mtDNA A10398G (A to G transition due to an amino acid substitution from Thr to Ala) was located in the ND3 gene that encodes one subunit constituting Complex I [7]. Complex I was the first enzyme in the mitochondrial respiratory chain and a key enzyme for energy production and a site for ROS production [23, 24]. Therefore, it is possible that 10398G may increase the performance of complex I. Thus, carriers of the 10398G allele would have enhanced capability for energy metabolism when the cell was either in a normal state or under abnormal oxidative stress conditions. An increase in complex I activity can result in an increase in ATP synthesis and protect against a range of PD-relevant biotoxins [25]. Thus, the carriers of the 10398G allele are at a lower risk for developing PD. In addition, the 10398G allele may induce more ROS production compared with 10398A. Complex I normally produces ROS during cellular activity [26]. However, when the complex I is enhanced, generation of ROS is decreased, alleviating oxidative stress [27]. SN tissues are susceptible to oxidative damage, and excessive oxidative stress over time may lead to the degeneration of SN neurons [28]. Therefore, the 10398G allele could play a role in determining neuronal protection to reduce the risk of developing PD. Further study is needed to verify the mechanism for its regulation on complex I.

Several reasons may help account for why the results between the Caucasian population and the Asian population are different. First, the A10398G polymorphism is differently distributed in the Caucasian population compared to the Asian population: the frequency of 10398G allele of mtDNA, which is found only 26% in Caucasian, is much higher in Asian population [29]. The negative result of the mtDNA A10398G polymorphism in PD in the Asian population may be due to the very high frequency of the 10398G allele in the Asian population. Second, because gene polymorphisms could influence the risk of developing PD through interaction with other genes or with some environmental toxins [30], an environmental difference between the Caucasian population and the Asian population may lead to the inconsistent results. Therefore, further well-designed studies with larger samples, particularly studies stratified by ethnic and gene-environmental interactions, should be performed to validate these results.

We are convinced that our meta-analysis has some special characteristics. Most importantly, compared with previous research, a larger sample size was used to estimate the effect, so our results are more reliable than the previous research results. In addition, according to a recent review on Parkinson's disease pharmacogenetics included all the meta-analysis published in PD [31], and A10398G was not included. Therefore this is apparently the first meta-analysis on this issue and this study is the first meta-analysis to demonstrate that the G allele of the mtDNA A10398G polymorphism does not appear to associated with PD risk in the Asian population, and it may be a potential protective factor of PD in the Caucasian population, which may encourage more research focused on the mtDNA A10398G polymorphism in different races. Last but certainly not least, we conducted a sensitivity analysis to estimate the effect of each study on the overall estimate, and the results suggested that our meta-analysis results were stable.

There are also some limitations of our meta-analysis. First, as PD is a multifactorial disease, the potential interactions of genetic-genetic or genetic-environmental factors may influence the process of PD. Second, in view of the relatively small sample size and the limited study number, especially in the subgroup of the Asian population where only three studies were included, the power used to detect the real difference between cases and controls may not be very strong. Finally, as a result of insufficient data, other possible variables, including age of onset, gender or smoking, for the association between A10398G and PD could not be evaluated.

In conclusion, our meta-analysis indicated that although the G allele of the mtDNA A10398G polymorphism has no significant relationship with PD in the Asian populations, it seems to be a potential protective factor of PD in the Caucasian population. Although a systematic investigation of the relationship between A10398G polymorphisms and the risk of PD could not be conducted because of the aforementioned limitations, it is important to gain a better understanding of the effect of A10398G polymorphisms on PD. Considering the limitations, investigations involving large case-control studies of different ethnicities should be conducted to clarify the possible roles of the mtDNA A10398G polymorphism in the aetiology of PD.

## MATERIALS AND METHODS

### Literature search

Two investigators independently searched the related literature from PubMed, Web of Science, Springer Link, EMBASE and EBSCO databases published up to March 2017. We used the following key words: (“mitochondrial DNA” or “A10398G”) and (“gene polymorphism” or “variant”) and (“Parkinson's disease” or “PD”). References of the eligible articles were also searched. Searching was restricted to human case-control studies.

### Inclusion and exclusion criteria

To be included, the studies must have met the following inclusion criteria: (1) case-control studies that studied the relationship between mitochondrial DNA A10398G and PD, (2) studies that included sufficient allele data for both patients and controls to calculate the odds ratio (OR) and 95% confidence interval (CI), (3) studies in which cases and controls were based on the same population, (4) studies that included participants diagnosed with PD according to the UK Parkinson's Disease Society Brain Bank criteria, and (5) full text studies written in English.

The excluded criteria included the following: (1) case reports, editorials, meta-analysis or reviews; (2) studies with insufficient information about the mitochondrial DNA A10398G; (3) family-based studies; (4) studies whose subjects were ethnic minorities; and (5) studies in which the allele distribution in controls did not meet Hardy-Weinberg equilibrium (HWE).

### Extraction

Two investigators independently selected the eligible articles and extracted the needed data. Inconsistencies between the two investigators were resolved by discussing with a third reviewer. The information extracted from each study included the first author's name, year of publication, ethnicity of the study population, and number of cases and controls for each allele.

### Quality assessment

The Newcastle-Ottawa Scale (NOS) [32] was used to estimate the quality of the eligible studies by two reviewers independently based on the following aspects: selection, comparability and exposure. The NOS scores of each article equal or greater than 6 stars indicated high quality.

### Statistical analysis

We used the Stata 12.0 software (Stata Corporation, College Station, TX, USA) to calculate all data analyses. In addition, we used the allele model to evaluate the association between mtDNA A10398G variant and PD risk. The OR and CI were used to assess the strength of the association between the variant and PD susceptibility. The pooled OR was measured using the *Z*-test. *P* < 0.05 was considered to demonstrate a statistically significant difference. Heterogeneity between studies was examined by the *Q*-test (meaningful value of *P* < 0.05) and I-square statistic tests (I^2^ < 50% shows little difference) [33]. The fixed effects model or the random effects model was selected according to the heterogeneity. If the *P*-value of the *Q*-test > 0.05 or I^2^ < 50%, the fixed effects model was selected. Inversely, the random effects model was applied [34]. We also performed subgroup analysis to seek reasons for heterogeneity. Stratified analysis was performed according to ethnicity and source of controls. Sensitivity analysis was conducted to assess the stability of the results. Potential publication bias was assessed by Begg's test and funnel plots. A *P*-value of Begg's test greater than 0.05 and articles with symmetrical distribution in the Begg's funnel plot were considered to have no significant publication bias [35].
